# ﻿High-level phylogenetic relationships within *Pezizomycotina* revisited

**DOI:** 10.3897/imafungus.16.153279

**Published:** 2025-06-20

**Authors:** Vadim Goremykin, Claudio Donati

**Affiliations:** 1 Unit of Computational Biology, Research and Innovation Centre, Fondazione Edmund Mach, Via Mach 1, 38098 San Michele all’Adige (TN), Italy Unit of Computational Biology, Research and Innovation Centre, Fondazione Edmund Mach San Michele all’Adige Italy

**Keywords:** Classification, Pezizomycotina, phylogenomics

## Abstract

Here, we re-examine the high level phylogeny of *Pezizomycotina* with special attention to the recently proposed phylogenomic hypothesis (Díaz-Escandón et al. 2022) that “morphologically hyperdiverse” *Candelariomycetes*, *Coniocybomycetes*, *Geoglossomycetes*, *Lichinomycetes*, *Sareomycetes* and *Xylonomycetes* (henceforth referred to as classes *sensu stricto* (*s.s.*)) should be united in a class *Lichinomycetes* (henceforth referred to as *Lichinomycetessensu lato* (*s.l.*)), based on their common origin. Our examination revealed that the orthology of the aligned character states in the data used to produce this result is questionable due to the presence of poorly-aligned, indel-rich vertical alignment partitions, missing data and heterogeneous sequences. Our analyses of a thoroughly curated phylogenomic dataset and its subset with reduced compositional heterogeneity indicated that the fungi included in the *Lichinomycetess.l.* form six independent lineages, of which two correspond to *Geoglossomycetess.s.* and *Candelariomycetess.s.* and others do not correspond to the taxonomic delimitations of the previously defined classes. Based on the results obtained here, we propose to revise the class *Lichinomycetes* to include *Lichinomycetess.s.*, *Coniocybomycetess.s.* plus some *incertae sedis* genera (*Caeruleum*, *Thelocarpon*, *Piccolia*, *Sarcosagium* and *Vezdaea*). In our analysis, *Xylona* (*Xylonomycetess.s.*) plus *Sarea* (*Sareomycetess.s.*) were found to form an early diverging lineage within the branch also subtending *Arthoniomycetes* plus *Dothideomycetes*, which warrants the conclusion to include these two genera in a single class, whereas *Symbiotaphrina*, initially assigned to *Xylonomycetess.s.*, was found to split off the tree backbone earlier and, thus, should be treated as a separate lineage.

## ﻿Introduction

*Pezizomycotina*, a large subphylum with a worldwide distribution that comprises 16 classes of filamentous fungi and some dimorphic yeast-like forms, account for approximately 98.2% of described *Ascomycota* species and about two-thirds of all fungal species (Spatafora et al. 2017). *Pezizomycotina* are so morphologically diverse that the difficulties in identification of homologous characters and controversies about their significance for systematics once prompted the decision to avoid all ranks above the ordinal level in working out the morphology-based classification of this group (Eriksson and Hawksworth 1993). In view of these difficulties, it has long been acknowledged that comprehensive classification of these fungi is impossible without molecular evidence (Taylor 1995; Lumbsch 2000). However, despite more than two decades of subsequent molecular phylogenetic research, a number of the higher order relationships of *Pezizomycotina* remain controversial amongst studies (e.g. Spatafora (2017); James et al. (2020); Shen et al. (2020)). For instance, the earliest diverging lineage within the subphylum was suggested to comprise *Orbiliomycetes* (Spatafora et al. 2006; Prieto and Wedin 2013) or *Pezizomycetes* (Schoch et al. 2009; Beimforde et al. 2014) or both classes (Shen et al. 2020). However, arguably, the most recalcitrant and least well-understood issue in the high level phylogeny of these fungi concerns the evolutionary affinity of the so-called six “orphan” classes of *Pezizomycotina*, under-represented in the public sequence databases – *Candelariomycetes*, *Coniocybomycetes*, *Geoglossomycetes*, *Lichinomycetes*, *Sareomycetes* and *Xylonomycetes*. To name some examples, *Geoglossomycetes* was suggested to be a sister to a clade subtending all the *Pezizomycotina* with the exception of the *Orbiliomycetes* and *Pezizomycetes* (Schoch et al. 2009; Carbone et al. 2017; Beimforde et al. 2020) or a sister to the *Lichinomycetes*+*Coniocybomycetes* branch (Melie et al. 2023) or a sister to *Symbiotaphrinales* only (Hashimoto et al. 2021). *Candelariomycetes* were placed within a cluster with *Lecanoromycetes* (Beimforde et al. 2020) or as a sister to the *Dothideomycetes*+*Arthoniomycetes* lineage (Nelsen et al. 2020) or as a sister to the *Xylonomycetes*+*Geoglossomycetes*+*Symbiotaphrinales* lineage (Hashimoto et al. 2021) or as a sister to the large branch uniting *Coniocybomycetes*, *Lichinomycetes*, *Lecanoromycetes*, *Arthoniomycetes*, *Dothideomycetes*, *Xylobotryomycetes* and *Eurotiomycetes* (Voglmayr et al. 2019). Recently, an intriguing phylogenomic hypothesis was put forward that all above mentioned six “orphan” *Pezizomycotina* classes form a morphologically hyperdiverse lineage sister to the clade of *Eurotiomycetes* and *Lecanoromycetes* (Díaz-Escandón et al. 2022). The authors expanded the previously narrowly defined class *Lichinomycetes* to include all six “orphan” classes and provided comments as to why this lineage was not identified before. Some of these comments pertain to absence of any evident, unifying feature of morphology that unites the members of the proposed lineage. Other are related to massive extinctions within the newly re-defined class, that contributed to apparent dissimilarity of the remaining survivors. In view of previous studies offering quite different views of *Pezizomycotina* phylogeny, this novel hypothesis, which was suggested to provide a new roadmap in the evolutionary studies of fungi (Arnold 2022), deserves further investigation.

There are objective factors that can negatively affect phylogenomic inference. It has long been noticed that genome-scale phylogenetic analyses also lead to an increase in errors in alignment construction (Philippe et al. 2011; Laurin-Lemay et al. 2012). This is because the huge amount of genomic/transcriptomic data has become difficult to handle manually. The error in phylogenetic inference increases with the alignment error (e.g. Ogden and Rosenberg (2006)), so increasing the amount of data used for phylogeny reconstruction does not necessarily results in increased accuracy of inference, especially if the accumulation of vast datasets is achieved at the expense of alignment quality (Philippe et al. 2011) and rigorous identification of orthologous genes (Altenhoff et al. 2019; Siu-Ting et al. 2019; Cheon et al. 2020). Additionally, the need to analyse very large datasets in a reasonable amount of time necessarily imposes hard limits on the complexity of the evolutionary models used in such analyses (Lartillot 2020; Pandey and Braun 2021). Errors in inference due to poor model fit do not go away with increase in sequence length (Kapli et al. 2020).

With that in mind, we revisited the high level phylogeny of *Pezizomycotina*, taking special care to avoid errors in data preparation and analysis. The results we obtained indicate that the cluster of *Candelariomycetes*, *Coniocybomycetes*, *Geoglossomycetes*, *Lichinomycetes*, *Sareomycetes* and *Xylonomycetes* (Díaz-Escandón et al. 2022) is a phylogenetic artefact and that the species included in the cluster in the above study represent six lineages with different phylogenetic affinities within the *Pezizomycotina*. Taxonomic composition of these lineages largely does not coincide with the delimitations of the above six classes. Here, we discuss implications of the results and observations obtained here for higher-order taxonomy of *Pezizomycotina* and for experimental design for phylogenomic studies.

## ﻿Methods

### ﻿Identification of orthologues

Annotated genome sequences and metagenome-assembled genomes (MAGs) of *Pezizomycotina* and the closest outgroup (*Jarrowia*) (115 in total) were downloaded as supplementary material provided by Díaz-Escandón et al. (2022) (available at https://doi.org/10.6084/m9.figshare.19558762). The annotated protein sequences were analysed to find orthologous gene families using OrthoFinder v. 2.5.4 (Emms and Kelly 2019). The sequences of the hierarchical orthologous groups (HOGs) that contained all the species were sampled from the alignments produced by Orthofinder and written in individual alignment files, one per each HOG. These alignments were trimmed with Gblocks (Castresana 2000) under the default parameters. The alignments of divergent sequences where Gblocks could not identify a single conserved block of sequences were discarded at this stage. Trees were built, based on the trimmed alignments using FastTree v. 2.1.11 (Price et al. 2010) under the default parameters. Based on these trees, a post-processing refinement of the alignments by removing sequences related through gene duplication events was performed employing Phylopypruner v.1.2.4 (https://gitlab.com/fethalen/phylopypruner) with the default parameters, specifying the “--min-support 0.9” option in order to collapse branches with an SH-like support value below 0.9, which is a conserved selection threshold, as defined in the original study (Guindon et al. 2010). Resulting trimmed alignments, wherein each of the 115 species was represented by a single sequence, were visually inspected to identify those with obvious similarity between in- and outgroup. This post-processing resulted in 200 alignments wherein each of the 115 species was represented by a single sequence. Examination of in- and output data of Phylopypruner revealed that 199 of these alignments were produced by excision of homologous gene copies in ≤ 4 species per alignment by Phylopypruner. One of these alignments were produced by excision of homologous gene copies in five species by Phylopypruner.

### ﻿Alignment preparation

The protein sequences contained in these 200 trimmed alignment files were fetched from the untrimmed alignment versions produced by Orthofinder and re-aligned with MAFFT v.7.397 (Katoh et al. 2005) under a thorough (LinSi) algorithm. These alignments were concatenated and the resulting data matrix (henceforth referred as to “alignment A”, publicly available in the Zenodo repository (https://doi.org/10.5281/zenodo.14945427)) was edited in the Seaview alignment editor (Gouy et al. 2010) as follows. A preliminary selection of blocks was done by the Gblocks programme embedded in the Seaview alignment editor, toggling options “allow gap positions within the final blocks” and “do not allow many contiguous non-conserved positions” on (these options are equivalent to -b5=h and -b3=4 command line options of the stand-alone Gblocks programme). Each resulting block of sequences was visually inspected and, if necessary, its borders were manually edited in Seaview in order to ensure that only unambiguously aligned vertical alignment partitions would be selected for phylogenetic inference. The concatenated alignment with a resulting site selection was subjected to a manual trimming of individual sequences in selected blocks with the purpose to substitute the sequence stretches in species that appeared to be clearly misaligned with the rest of the species with gaps. Such non-homologous sequence stretches resulting, for example, from frameshifts due to poor sequence quality or annotation errors in individual sequences, were reported to detrimentally affect phylogeny reconstruction (Whelan et al. 2018). The resulting 83,408 position-long set of edited vertical sequence blocks was saved in a file, henceforth referred to as the “alignment B”. The alignment, containing 1.3% of gaps, is publicly available in the Zenodo repository (https://doi.org/10.5281/zenodo.14945427).

### ﻿Reduction of compositional heterogeneity

In the alignment B, 55% of sequences (63 sequences in total) failed at 0.05 significance level the chi2 test of compositional homogeneity as implemented in IQ-TREE v. 2.3.6 (Nguyen et al. 2015). Since compositional bias amongst the sequences was shown to lead to errors in phylogeny reconstruction (Jermiin et al. 2004; Gruber et al. 2007; Blanquart and Lartillot 2008; Duchêne et al. 2017; Goremykin 2023), a sampling of sites with the reduced compositional heterogeneity from the above alignment was performed with the BMGE programme (Criscuolo and Gribaldo 2010). The thorough search algorithm as implemented in the program (“-s YES” option), allowed us to identify the 70,401 position-long vertical partition of the alignment B wherein all the sequences passed the above mentioned chi2 test, as well as all matched-pairs tests of marginal symmetry (Stuart 1955), performed using the SymTest programme (Ababneh et al. 2006) at 0.05 significance level. These data (henceforth referred to as the “alignment C”) is publicly available in the Zenodo repository (https://doi.org/10.5281/zenodo.14945427).

### ﻿Alignment quality assessment

In order to compare alignment quality, two approaches were used – one that identifies and removes stretches of contiguous alignment positions with low conservation (as implemented in Gblocks) and another one that trims divergent sequences borne on very long branches (as implemented in Phylopypruner). Gblocks-based alignment trimming was performed, based on the 653,307 position-long concatenated alignment of 1,292 protein alignment files that were provided as supplementary material by Díaz-Escandón et al. (2022) using -b5=h -b3=4 command line options that were used for preliminary selection of blocks for the alignment B. The alignment B file was subject to the analogous trimming procedure for the purpose of comparison. In order to assess the quality of individual protein alignments, both alignments mentioned above were split on to the alignment partitions corresponding to the individual proteins and each partition file was subject to the Gblocks-based alignment trimming with the above parameters.

Trees were built with the IQ-TREE v. 2.3.6 programme under the LG+I+G model, based on each of 1,292 above-mentioned individual protein alignments used in Díaz-Escandón et al. (2022). These trees with the corresponding alignments were trimmed using Phylopypruner with --trim-lb 5 flag in order to remove sequences with a branch length that is longer than five standard deviations of all branch lengths in each tree. Each of the 200 alignments of the individual proteins sampled from the alignment B was trimmed with Phylopypruner as described above to generate analogous statistics for the purpose of comparison.

### ﻿Phylogenetic inference: attempt to reproduce *Lichinomycetess.l.* clade

In order to check the reproducibility of the results in Díaz-Escandón et al. (2022) with our data, we first attempted to evaluate the support for their hypothesis under site-homogeneous models. Trees were built under LG and WAG models with rate heterogeneity amongst sites modelled via “CAT” approximation as a workaround for the computationally intense gamma model using: i) the approximate Maximum Likelihood method of inference as implemented in FastTree v. 2.1.11 (Price et al. 2010) and ii) the Maximum Likelihood method of inference as implemented in RaxML v. 8.2.9 (Stamatakis 2014), based on the alignments A, B and C. Maximum Likelihood trees were also built with the similar LG+G and WAG+G models with the help of the IQ-TREE v. 2.3.6 programme, based on the alignments A, B and C. Considering the extent of reproducibility of the *Lichinomycetess.l.* clade in these experiments, the quality of the tree topologies obtained, based on the alignment A, was assessed. Following the protocol provided in the original paper on FastTree (Price et al. 2010), the branch lengths of each tree were re-optimised under the same empirical substitution matrix used for its construction and rate heterogeneity amongst sites modelled with the discrete gamma model with four categories of rates in RaxML in order to obtain comparable likelihood scores. The statistical significance of competing phylogenetic hypotheses obtained, based on the alignment A was assessed with the approximately unbiased (AU) test (Shimodaira 2002). For this purpose, the trees with the branch lengths adjusted under the LG+G model were concatenated in one file and the trees with the branch lengths adjusted under the WAG+G model were concatenated in another file. Site-wise log-likelihood values were separately calculated using RaxML, based on each file under the specification of the corresponding model. The resulting two RaxML output files were analysed with Consel (Shimodaira and Hasegawa 2001) with the default settings to calculate p-values for the AU test, based on 10,000 RELL replicates.

### ﻿Phylogenetic inference under a site-heterogeneous model

A relative fit of: i) the homogeneous substitution models assuming empirical matrices of substitution rates and ii) a GTR-based model that utilised a substitution rate matrix derived from the curated alignment B, to the alignment B was estimated with the ModelFinder pipeline (Kalyaanamoorthy et al. 2017) implemented in IQ-TREE. An analogous experiment was conducted, based on the alignment C with the reduced compositional bias amongst sequences. In both experiments, it was consistently observed that, under a model composition that was fixed with the exception of the substitution matrix, the use of the GTR-based substitution matrix resulted in a better model fit under the Bayesian Information Criterion (BIC). Based on this observation, we chose to infer *Pezizomycotina* phylogeny under a site-heterogeneous model using a GTR-based substitution matrix and Dirichlet processes for modelling: i) sites-specific frequency profiles and ii) the distribution of relative rates of substitution across sites (CAT+GTR+D).

**Figure 1.**
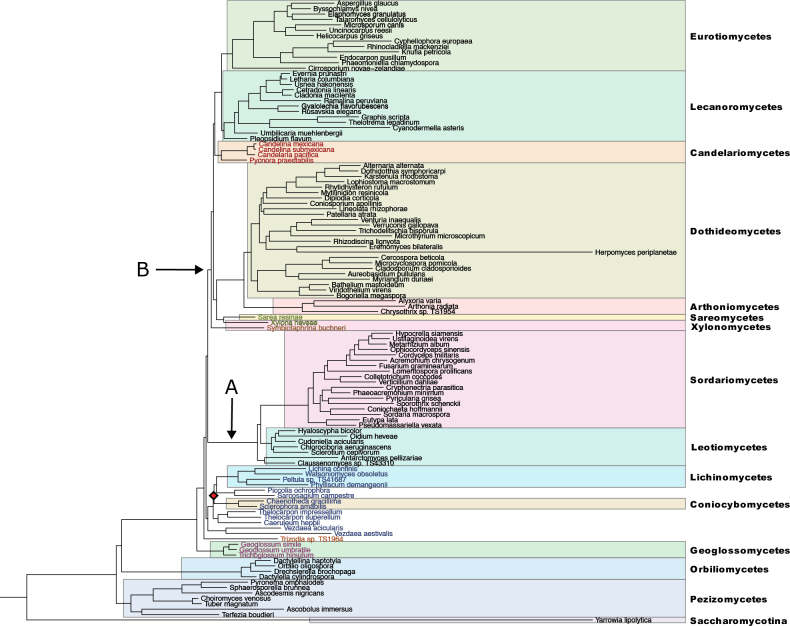
The consensus phylogenetic tree showing the relationship between the major lineages of *Pezizomycotina* as inferred under a CAT+GTR+D model. The clade marked with a diamond received 0.5 posterior probability support (PP). All other clades received maximum posterior probability support (1 PP). The taxonomic assignment of the species to the classes of *Pezizomycotina*, shown to the right of the phylogram, follows the original higher order classification of *Pezizomycotina* discussed in Díaz-Escandón et al. (2022) and shown in their fig. 1. *Yarrowialipolytica* (*Saccharomycotina*) is shown as outgroup. The six lineages, marked in the figure by red, green, brown, blue, orange and violet colour of their member species, mentioned in the main text, were assigned to *Lichinomycetess.l.* by Díaz-Escandón et al. (2022). The Clades A and B, mentioned in the main text, are indicated with the arrows marked A and B, respectively.

A chain was run, based on the alignments B and another chain was run, based on the alignment C in PhyloBayes v.4.1 (Lartillot et al. 2009) to sample, for each chain, 3000 cycles under a CAT+GTR+D model. After checking the extent of the “burn-in” zones for each chain, the consensus trees were built, discarding incrementally increasing number of cycles, to determine a point at which the mean discrepancies in clade support values (meandiff value) calculated from both chains would be minimised. The consensus phylogenetic tree (Fig. 1) was built, based on the last 1700 cycles sampled from each chain (3400 cycles in total), after a plateau of the minimum meandiff values (0.00440529), corresponding to a minimum difference in individual tree topologies, was reached.

## ﻿Results

### ﻿Alignment quality checks

Trimming with Gblocks resulted in removal of 0.7% of the alignment columns from the alignment B used in this study. An analogous value obtained, based on the 653,307 position-long concatenated alignment of the 1,292 proteins selected for phylogeny inference in Díaz-Escandón et al. (2022), was 36%. In the series of site-trimming experiments aimed at the assessment of the quality of the alignment partitions corresponding to the individual proteins in the above alignment, we observed that 109, 165, 225, 321 and 422 of these partitions were shortened by more than 80, 70, 60, 50 and 40 percent, respectively. The analogous values calculated for 200 individual protein alignments used to build the alignment B were all zero.

Trimming with Phylopypruner resulted in removal of 108277 individual protein sequences (18.8% of the total number of sequences) from the individual protein alignment files used by Díaz-Escandón et al. (2022) for phylogeny reconstruction. In an analogous experiment, based on the 200 individual trimmed protein alignments used to build the alignment B, Phylopypruner deleted 88 individual gene sequences (0.38% of the total number of sequences). The programme removed more than 50% and 25% of sequences initially present in the individual protein alignments from, respectively, 153 and 353 individual alignment files used for phylogeny reconstruction by Díaz-Escandón et al. (2022) The analogous values calculated, based on the individual trimmed alignment files used here, were all zero.

Spot-checking gene trees with very long branches and blasting corresponding sequences vs. a local BLAST database containing sequences classified into orthologous groups by Orthofinder allowed us to identify 46 individual protein alignments used in Díaz-Escandón et al. (2022), wherein species of *Pezizomycotina* shown in the phylogram (Fig. 1) in the above paper were represented by paralogous sequences belonging to distinct orthologous groups as classified by Orthofinder. It should be noticed that this number is likely to be underestimated, since the sequences used for blasting were partial sequences taken from the alignments, which were automatically curated in the above study, which made an assignment of the BLAST query sequences to orthologous groups difficult in a large number of cases.

### ﻿Reproduction of the *Lichinomycetess.l.* cluster reported by Díaz-Escandón et al. (2022)

With our data, we were able to recover the clade subtending all the species assigned to *Lichinomycetess.l.* by Díaz-Escandón et al. (2022). This result was obtained under the approximate Maximum Likelihood method of inference (as implemented in FastTree) with the LG and WAG models and a rate heterogeneity amongst sites modelled via “CAT” approximation, based on the untrimmed alignment assembled prior to removal of poorly-aligned alignment regions (alignment A). The internal topology of the above clade comprising 24 species was fully resolved in both experiments and was not in conflict with the hypothesis of relationship of these species within the *Lichinomycetess.l.* cluster as presented in Díaz-Escandón et al. (2022) in their fig. 1. An attempt to recover a branch subtending the same 24 species under the Maximum Likelihood method of inference, based on the alignment A under analogous LG+”CAT” and WAG+”CAT” models (as implemented in RaxML) and LG+G and WAG+G models (as implemented in IQ-TREE) were unsuccessful. Analogous attempts to recover the branch subtending *Lichinomycetess.l.* with the FastTree, RaxML and IQ-TREE programmes utilising the empirical LG and WAG substitution matrices, based on the curated alignments B and C failed. Comparison of log-likelihood values for the tree topologies recovered from the alignment A with FastTree, RaxML and IQ-TREE using the LG substitution matrix revealed that the value for the topology supporting the hypothesis of monophyletic *Lichinomycetess.l.* was the lowest as evaluated under an LG+G model (Table 1). Analogous evaluation of the results obtained under the WAG substitution matrix also indicated the lowest likelihood value for the topology supporting *Lichinomycetess.l.* (Table 1). Within both sets of tree topologies, one recovered under the LG matrix and the other recovered under the WAG matrix, the approximately unbiased (AU) test rejected the tree topologies supporting the hypothesis of monophyletic *Lichinomycetess.l.* at the significance level P = 0.05.

**Table 1.** The likelihood values for the the tree topologies built, based on the alignment A under homogeneous models assuming LG and WAG exchangeability matrices with the branch lengths re-optimised in RaxML employing the discrete gamma model with four categories of rates.

Full evolutionary model	Likelihood value
LG+G & FastTree tree	-15986782.257036
LG+G & RaxML tree	-15983428.581759
LG+G & IQ-TREE tree	-15983415.842263
WAG+G & FastTree tree	-16059720.686598
WAG+G & RaxML tree	-16056399.348728
WAG+G & IQ-TREE tree	-16056399.348913

Note: The full evolutionary modes (model+tree combinations) used to calculate the likelihood values in RaxML are shown in the first column. The likelihood values obtained under the full evolutionary models assuming an LG+G model component were calculated for the tree topologies inferred using FastTree, RaxML and IQ-TREE under LG+”CAT”, LG+”CAT” and LG+G substitution models, respectively. The likelihood values obtained under the full evolutionary models, assuming a WAG+G model component, were calculated for the tree topologies inferred using FastTree, RaxML and IQ-TREE under WAG+”CAT”, WAG+”CAT” and WAG+G substitution models, respectively.

### ﻿Results of site-heterogeneous analyses

In the consensus tree, presented here in Fig. 1, all the internal branches, except one, marked with a diamond, were supported by the maximum posterior probability (PP) value (1). *Pezizomycetes* was resolved as the earliest diverging clade within *Pezizomycotina* and the clade subtending *Orbiliomycetes* branched off next. A notable feature of the consensus tree is that the 24 species forming the *Lichinomycetess.l.* cluster in the phylogeny reconstructed by Díaz-Escandón et al. (2022) (shown here in Suppl. material 1: fig. S1, with the same annotation and highlighting colour scheme as in Fig. 1 for comparison) form six independent lineages (marked by red, green, brown, blue, orange and violet colour of their member species). In our reconstructed phylogeny (Fig. 1), one of these lineages encompassing species originally assigned to *Geoglossomycetess.s.* (highlighted in violet) forms an early-diverging clade that split off the main stem of the tree after branching off of *Orbiliomycetes*. Another lineage, *Trizodia* sp. TS1964, (highlighted in orange) splits off next, followed by the lineage subtending 13 species (highlighted in blue) also assigned to *Lichinomycetess.l.* in Díaz-Escandón et al. (2022). These species were originally placed in *Coniocybomycetess.s.*, *Lichinomycetess.s.* and several genera described as *incertae sedis* (*Caeruleum*, *Thelocarpon*, *Piccolia*, *Sarcosagium* and *Vezdaea*). The crown group of *Pezizomycotina* in our reconstructed phylogeny consists of two sister lineages, one with *Leotiomycetes* and *Sordariomycetes* (shown with an arrow marked “A” and, henceforth, referred to as the “Clade A”) and another with *Arthoniomycetes*, *Dothideomycetes*, *Eurotiomycetes*, *Lecanoromycetes* and a number of genera assigned to *Lichinomycetess.l.* by Díaz-Escandón et al. (2022) (shown with an arrow marked “B” and henceforth referred to as the “Clade B”). Within Clade A, the species assigned to *Leotiomycetes* appear as a paraphyletic assemblage. The branch subtending *Leotiomycetes* species *Antarctomycespellizariae* + *Claussenomyces* sp. TS43310 appears at the earliest diverging position, followed by monophyletic *Sordariomycetes* being sister to the rest of *Leotiomycetes* included in our analysis. Within Clade B, *Symbiotaphrina buchneri* (highlighted in brown), a member of *Lichinomycetess.l.* sensu Díaz-Escandón et al. (2022), originally assigned to *Xylonomycetes*, was resolved as a distinct branch. Other species in Clade B fall into two monophyletic subclades, each containing members of the hypothesised *Lichinomycetess.l.* (Díaz-Escandón et al. 2022) as the earliest diverging lineages. In one of these subclades, the basal-most lineage encompassing the species highlighted in red in Fig. 1 and originally assigned to *Candelariomycetess.s.* appears as sister to the *Eurotiomycetes*+*Lecanoromycetes* cluster. In the other subclade, the basal-most lineage of *Xylonaheveae* (*Xylonomycetess.s.*) plus *Sarearesinae* (*Sareomycetess.s.*), highlighted in green, was resolved as sister to the branch subtending *Arthoniomycetes* plus *Dothideomycetes*. A representative of the genus *Herpomyces*, initially assigned to the class *Laboulbeniomycetes*, (*Herpomycesperiplanetae*) branched off from within the radiation of *Dothideomycetes.* Additionally, *Cirrosporiumnovae-zelandae*, a single representative of the class *Xylobotryomycetes* in our reconstructed phylogeny, was recovered as an early-diverging lineage of *Eurotiomycetes*.

## ﻿Discussion

Character-based methods of phylogeny reconstruction, such as Maximum Likelihood and Bayesian Inference, implicitly assume that character states in alignment columns have descended from common ancestral character states. Presence of fast-evolving sequence regions, missing data, divergent sequences due to paralogy or elevated substitution rate in the input data can lead, by disrupting alignment and/or introducing non-homologous sequence material, to violation of the above assumption and to errors in phylogenetic inference. This consideration is all the more important in the age of phylogenomics, because the need to analyse very large datasets often precludes manual control of data quality and forces scientists to accept errors generated by automated data curation pipelines. Moreover, computational time limitations associated with such analyses often force specialists to make a choice in favour of fast inference methods and simplified substitution models.

Considering that the availability of vast quantities of data can give rise to both major benefits and serious risks, we re-examined the hypothesis of monopyly for the six “orphan” classes of *Pezizomycotina* (Díaz-Escandón et al. 2022), trying to retain benefits and reduce risks. First, we decided not to use the results of the orthology assignment from the above paper — which were based on the BUSCO pipeline — due to poor alignment quality, obvious absence of input data quality control (as evident, for example, by inclusion of an alignment with only eight sequences into phylogenetic analyses) and absence of description of an algorithm for selection of sequences amongst multiple paralogous copies for downstream phylogenetic analysis. It should be noted that efficiency of the BUSCO software is known to be highly dependent on the underlying BUSCO dataset which should be chosen in such a way as to match and sufficiently well represent the taxon under study (Rödelsperger 2021; Wu et al. 2023). Even in the case of a perfectly well selected BUSCO database, multiple gene copies can be expected in up to 10% of the species per a BUSCO marker (Seppey et al. 2019). This number can be higher for the data used in Díaz-Escandón et al. (2022). Their gene sampling was derived from the predefined gene set from the dikarya_odb9 BUSCO database (Creation date: 13-02-2016, number of species: 75, number of BUSCOs: 1312). The database contains 37 species of *Pezizomycotina* (49% of all species), sampled from six *Pezizomycotina* classes. This sampling does not match the sampling of *Pezizomycotina* (395 species, 16 *Pezizomycotina* classes) for which phylogenetic relationships are inferred in the above study. Thus, in order to prepare alignment data for analysis, one obstacle that must be overcome is dealing with multiple paralogous gene copies.

In order to avoid errors in orthologue detection, we employed a dedicated orthologue inference pipeline (Orthofinder) for the phylogenetic analysis (which can be employed to detect single copy orthologues for any group of organisms) based on the uniform functional annotation of 115 genomes (Díaz-Escandón et al. 2022) selected by the authors to represent “all classes and lifestyles in *Pezizomycotina*”. The phylogenetic relationships amongst these 115 species (Díaz-Escandón et al. (2022), Fig. 1), represent the main result of the study.

Our selection of alignment and manual alignment curation steps was based on the premise that, in the preparation of phylogenomic data encompassing many genes, it is preferable to omit unreliably aligned regions and sequences to increase confidence in the orthology of the remaining character states to be used in the downstream analyses. To increase confidence that results obtained, based on the curated alignment, represent species phylogeny and not a non-phylogenetic compositional signal, we included in our analyses a dataset with reduced compositional heterogeneity amongst sequences.

Generally speaking, the implicit purpose of increased site sampling in phylogenomic analysis is to reach the zone of consistency in phylogenetic inference that can be expected under the correct model. Practically, although the models chosen for analysis of empirical data are never correct, the substitution models that consider heterogeneity at the site level (i.e. site-heterogeneous models that partition data at the level of sites, as opposed to at the level of loci) have been shown to provide a better approximation of the real evolutionary process than site-homogeneous models in most cases (Philippe et al. 2011; Wang et al. 2019) and to reduce systematic errors in phylogeny reconstruction (Meiklejohn et al. 2016; Schwentner et al. 2018). It has also been recently demonstrated that summarising individual gene trees in a species tree, as it was done by Díaz-Escandón et al. (2022), is statistically consistent with sequence data only under an unrealistic assumption of an infinite number of sites per gene (Roch et al. 2019). In realistic experimental settings, such analyses (partitioned concatenation and tree summary, used in Díaz-Escandón et al. (2022)) were shown to multiply small-sample LBA biases in phylogenetic inference (Roch et al. 2019). Citing these findings, in a follow-up methodological paper (Mirarab et al. 2021), the authors urge caution in using this methodology.

There are reasons to suspect error(s) in the cladogram (Fig. 1), presented in Díaz-Escandón et al. (2022). The results supporting the hypothesis of the common origin of *Candelariomycetess.s.*, *Coniocybomycetess.s.*, *Geoglossomycetess.s.*, *Lichinomycetess.s.*, *Sareomycetess.s.* and *Xylonomycetess.s.* were obtained here only based on the alignment A which was produced prior to alignment quality assessment and removal of poorly-aligned alignment regions. These results were rejected by the AU test. All analyses performed here, based on the curated alignment and its partition with reduced compositional heterogeneity amongst species, did not support the hypothesis of the common origin of the above-mentioned *Pezizomycotina* classes. Thus, we consider the *Lichinomycetess.l.* clade to be an artefact which can be attributed to the poor quality of alignment and/or limitations in analysis methodology. We consider the results of our site-heterogeneous analyses (Fig. 1) to provide a more accurate representation of the true phylogeny of *Pezizomycotina*.

Some aspects of the phylogenetic relationships of the “orphan” *Pezizomycotina* classes inferred here were reported in previous phylogenetic studies. The placement of *Geoglossomycetes* as a sister to all the *Pezizomycotina* with the exception of the *Orbiliomycetes* and *Pezizomycetes* (Fig. 1) was initially inferred in Schoch et al. (2009), who erected this taxon and later in Carbone et al. (2017), Prieto et al. (2019) and Beimforde et al. (2020). The close phylogenetic affinity of *Coniocybomycetess.s.* to *Lichinomycetess.s.*, which together form a 13-member cluster with a number of *incertae sedis* taxa in our reconstructed phylogeny (Fig. 1), was also previously supported in Prieto et al. (2019) and Díaz-Escandón et al. (2022). The results reported here and in Díaz-Escandón et al. (2022) support the placement of *Herpomycesperiplanetae*, initially assigned to the class *Laboulbeniomycetes*, to *Dothideomycetes* and indicate that *Cirrosporiumnovae-zelandae*, initially assigned to *Xylobotryomycetes*, is an early-diverging lineage of *Eurotiomycetes*. It is evident from our results that *Xylonaheveae* (*Xylonomycetess.s.*) and *Sarearesinae* (*Sareomycetess.s.*) form a lineage sister to *Arthoniomycetes* plus *Dothideomycetes*, which lends strength to the previous suggestion to synonymise *Sareomycetes* with *Xylonomycetes* (Hashimoto et al. 2021). At the same time, the phylogenetic affinity of *Symbiotaphrina* to *Xylonomycetes*, reported in Díaz-Escandón et al. (2022) is rejected by Hashimoto et al. (2021) and in our analyses. According to our knowledge, the placement of *Symbiotaphrina* as the earliest diverging lineage in the branch B (Fig. 1) was not recovered in previous analyses. Additionally, according to our knowledge, the inferred placement of *Candelariomycetes* as sister to *Eurotiomycetes*+*Lecanoromycetes* (Fig. 1), was not previously reported.

In accordance with the results obtained, we propose the following nomenclatural changes: (1) to include the members of the clade, comprising *Lichinomycetess.s.*, *Coniocybomycetess.s.* plus *incertae sedis* genera *Caeruleum*, *Thelocarpon*, *Piccolia*, *Sarcosagium* and *Vezdaea*, (Fig. 1, highlighted in blue) in a single class, *Lichinomycetes*, referring to it by the oldest name contained within it; (2) to assign members of the clade subtending *Xylonaheveae* (*Xylonomycetess.s.*) and *Sarearesinae* (*Sareomycetess.s.*) to *Xylonomycetes*, referring to it by the oldest name contained within the clade; and (3) to exclude *Symbiotaphrina buchneri* from *Xylonomycetes*.

Taken together, the analyses presented in this study provide new insights on the general pattern of diversification in the subphylum *Pezizomycotina* and phylogenetic affinities of the “orphan” *Pezizomycotina* classes. These were obtained based on a large, well-curated dataset under, arguably, a realistic site-heterogeneous substitution model. In the presented study, however, taxon sampling for the “orphan” classes is still limited, so that there are long single-species branches (e.g *Symbiotaphrina buchneri* and *Trizodia* sp. TS1964) in the tree. Therefore, the validity of these results should be checked in the future with larger sample of taxa representing these lineages.

## ﻿Conclusion

In this study, we prepared a large and thoroughly curated phylogenomic dataset in order to investigate high level phylogenetic relationships within *Pezizomycotina*. Our phylogenomic analyses under a site-heterogeneous model indicate that recently proposed re-classification of *Pezizomycotina* assuming merging *Candelariomycetess.s.*, *Coniocybomycetess.s.*, *Geoglossomycetess.s.*, *Lichinomycetess.s.*, *Sareomycetess.s.* and *Xylonomycetess.s.* in a single class, *Lichinomycetess.l.*, is incongruent with phylogenetic relationships amongst these lineages. The results obtained here indicate that the fungi included in the *Lichinomycetess.l.* form six independent lineages, of which two correspond to *Geoglossomycetess.s.* and *Candelariomycetess.s.* and others do not correspond to the taxonomic delimitations of the previously defined classes. Based on the results obtained, we propose to re-define the class *Lichinomycetes* to include *Lichinomycetess.s.*, *Coniocybomycetess.s.* plus some *incertae sedis* genera (*Caeruleum*, *Thelocarpon*, *Piccolia*, *Sarcosagium* and *Vezdaea*). Our analysis revealed that Xylona (*Xylonomycetess.s.*) and Sarea (*Sareomycetess.s.*) form a basal lineage within the branch that also includes *Arthoniomycetes* and *Dothideomycetes*. This justifies the conclusion that these two genera belong to a single class. On the other hand, *Symbiotaphrina*, which was originally assigned to *Xylonomycetess.s.*, was found to split off from the phylogenetic tree earlier and should, therefore, be treated as a separate lineage.

## ﻿Additional information

### Author ORCIDs

Vadim Goremykin https://orcid.org/0000-0002-1316-7366

Claudio Donati https://orcid.org/0000-0001-8688-1651

### Data availability

All alignments analysed during this study are available at Zenodo (https://doi.org/10.5281/zenodo.14945427).

## Supplementary materials

Supplementary material 1 Reconstructed phylogeny for 115 species as shown in figure 1 from Díaz-Escandón et al. (2022) Data type pdf Explanation note The figure was made based on the tree “6_fossil.treefile” in Newick format provided as Supplementary Material by the authors. https://binary.pensoft.net/file/1358149This dataset is made available under the Open Database License (http://opendatacommons.org/licenses/odbl/1.0/). The Open Database License (ODbL) is a license agreement intended to allow users to freely share, modify, and use this Dataset while maintaining this same freedom for others, provided that the original source and author(s) are credited.Vadim Goremykin, Claudio Donati
