# Supplementary material for: ﻿High-level phylogenetic relationships within Pezizomycotina revisited
Source: IMA Fungus. 2025 Jun 20;16:e153279. doi: 10.3897/imafungus.16.153279 (PMC12238968; doi:10.3897/imafungus.16.153279)
Supplement: Supplementary material 1 — Reconstructed phylogeny for 115 species as shown in figure 1 from Díaz-Escandón et al. (2022) [file imafungus-16-e153279-s001.pdf]

Supplementary Figure 1. Reconstructed phylogeny for 115 species as shown in Figure 1 from Díaz-Escandón et al. (2022). The figure was made based on the tree “6_fossil.treefile” in Newick format provided as Supplementary Material by the authors.

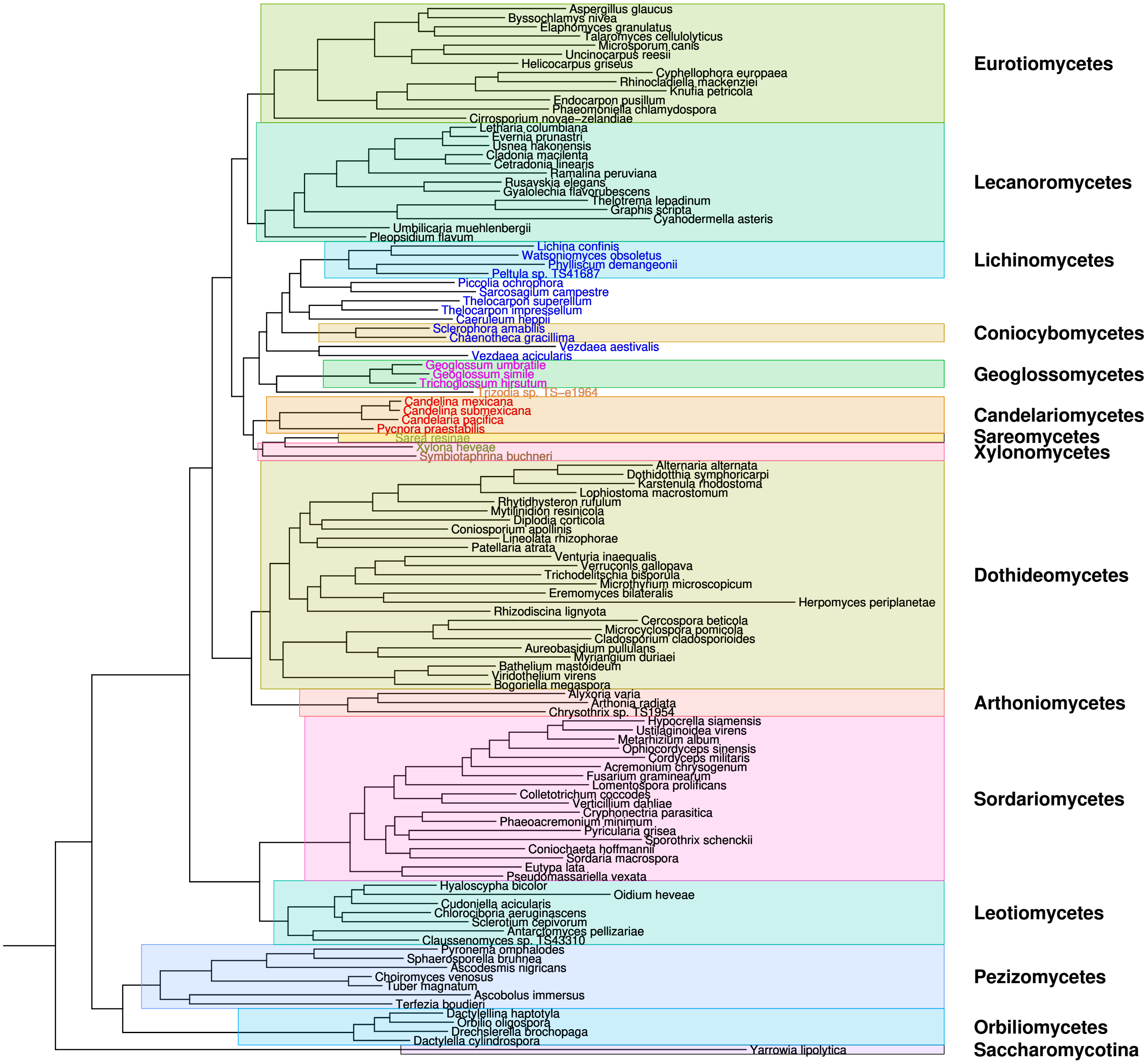

Note: The taxonomic assignment of the species to the classes of *Pezizomycotina*, shown to the right of the phylogram, color-coding scheme used for highlighting the names of species and different clades, and, where possible, the order of species in subclades, correspond to those used in Figure 1 in the presented study in order to facilitate comparison.
